# Imaging & identification of malaria parasites using cellphone microscope with a ball lens

**DOI:** 10.1371/journal.pone.0205020

**Published:** 2018-10-04

**Authors:** Temitope E. Agbana, Jan-Carel Diehl, Fiona van Pul, Shahid M. Khan, Vsevolod Patlan, Michel Verhaegen, Gleb Vdovin

**Affiliations:** 1 Delft Center for Systems and Controls, Delft University of Technology, Delft, The Netherlands; 2 Design for Sustainability, Industrial Design Engineering, Delft University of Technology, Delft, The Netherlands; 3 Parasitology and Immunologyparasitology Group, Leiden University Medical Center, Leiden, The Netherlands; 4 Flexible Optical BV, Rijswijk, The Netherlands; Texas A&M University, UNITED STATES

## Abstract

We have optimized the design and imaging procedures, to clearly resolve the malaria parasite in Giemsa-stained thin blood smears, using simple low-cost cellphone-based microscopy with oil immersion. The microscope uses a glass ball as the objective and the phone camera as the tube lens. Our optimization includes the optimal choice of the ball lens diameter, the size and the position of the aperture diaphragm, and proper application of immersion, to achieve diagnostic capacity in a wide field of view. The resulting system is potentially applicable to low-cost in-the-field optical diagnostics of malaria as it clearly resolves micron-sized features and allows for analysis of parasite morphology in the field of 50 × 50 *μ*m, and parasite detection in the field of at least 150 × 150 *μ*m.

## Introduction

Malaria is a life threatening disease prevalent in tropical and subtropical countries with high mortality and significant economic loss. Based on World Health Organization (WHO) report, 429,000 death cases were attributed to malaria in the year 2015. 212 million new cases of malaria worldwide was reported in the same year and about 3.2 billion people remain at risk of malaria globally [1].

Development of rapid diagnostic test (RDT) kits has enabled reliable detection of malaria infections particularly in remote areas with limited access to quality microscope services [2, 3]. However, RDTs performance have been reported in literature [4, 5] to degrade in tropical areas. Detection capabilities is low in sensitivity and specificity as compared to conventional diagnostic methods. Its current sensitivity threshold is reported to be greater than 100 parasite/*μ*l of blood and as such not sensitive enough to detect early-stage infections. Polymerase Chain Reaction (PCR) molecular detection methods are proven to be excellent diagnostics approaches with high efficacy. The cost of PCR equipment and the need for specialized trained personnel however restricts its usability to standard and sophisticated clinical laboratory settings. Microscopic examination of thick and thin blood smear still remains the recommended gold standard for clinical diagnosis of malaria [6, 7]. Widespread application and availability of conventional microscopy in remote low resource settings where malaria is prevalent is limited by (i) high cost, (ii) bulkiness of equipment, (iii) shortage of skilled personnel and (iv) lack of required equipment maintenance skills. Leveraging on the expansion in global cellphone network coverage, advances in cellphone imaging capabilities and computational power, simple high resolution diagnostic instrument useful in the global fight against malaria disease are fast becoming realizable. Optical techniques used in the design of cell-phone microscopy are based on (1) external optical attachment (2) on-lens design analysis and (3) on-chip optical design methodology.

Breslauer et al [8] implemented the external attachment technique by redesigning a standard microscope and attaching it to a cell-phone. The optical train attachment consist of a 60 × achromat objective and standard eyepiece to achieve a spatial resolution of 1.2 *μm*. Although the authors demonstrated the application of the system in the imaging and analysis of malaria infected blood smear, the use of conventional microscope optics and the fabrication of a bulky attachments increases the cost, complexity and required maintenance skills of the instrument. To circumvent the disadvantages of the bulky optical attachment, on-lens optical design techniques offer a relatively simple and low-cost alternative. Using this technique, a refractive optical element is directly attached to the camera lens of a cell-phone. Smith et al [9] reports on using a single ball lens attachment to a cell-phone for successful diagnosis of iron deficiency and sickle cell anemia in a blood smear. The imaging capability of same technique in the detection of soil helminths in stool sample using a single 3 mm ball lens has been demonstrated in [10]. With this technique, achievable spatial resolution is limited by the aberration of the attached optics. As a result, imaging of malaria parasite within the red blood cell was difficult to realize. Using a reversed mobile phone camera lens attachment, a larger field of view with unity magnification and a spatial resolution < 5 *μm* is reported in [11]. Detection of soil-transmitted helminth eggs in stool sample and imaging of red blood cells have been presented in their paper. Same technique with improved spatial resolution used for detection of *Shistosomiasis haemtobium* infection on the field is reported in [12].

On-chip technique for holography based microscopy demonstrated by researchers in University of California (UCLA) requires major hardware modification [13, 14]. A fabricated holographic platform is used as a replacement for the original cell-phone objective lens. With this technique, a high field of view without loss in spatial resolution was reported. However, reconstructing a standard image from the recorded fringe patterns is computationally demanding. Furthermore, holographic microscopy requires a small sample to sensor distance [15–17]. This makes its application for imaging of blood smear and biological tissues a bit more cumbersome. It’s application in the imaging of red and white blood cells as well as Giardia lambia cyst has been experimentally validated.

Mobile-based optical polarization imaging device reported in [18] detects hemozoins crystals in infected blood smears. Integrated optics include low cost plastic lens assembly which increases system aberration and complexity. Since hemozoins crystals are formed at the later stage of the ring form of malaria parasite, imaging of the early ring trophozoite cannot be demonstrated.

Taking advantage of the low-cost cell-phone with high pixel resolution sensors, advances in low-power light-emitting diodes (LEDs) and 3-D printing technologies, a battery powered cell-phone based platform has been developed for field use. Optimized for use with immersion medium, our diagnostic instrument provides images with the morphology of the parasite at the early ring trophozoites and other mature stages of the parasite’s developmental cycle. To the best of our knowledge, this is the first practical demonstration of imaging and morphological identification of malaria parasite using immersion based on-lens optical design techniques. This constitutes a major difference between our work and existing research works with similar optical design methodology. Giemsa staining is a simple protocol where blood smears are immersed in a staining solution containing Azure B and Eosin Y and then rinsed in with water. As the stains are chemically stable, low cost and do not require access to laboratory equipment, they can be rapidly deployed in low resource settings where access to efficient clinical laboratory infrastructure is unavailable.

## Design and performance

**Fig 1:**
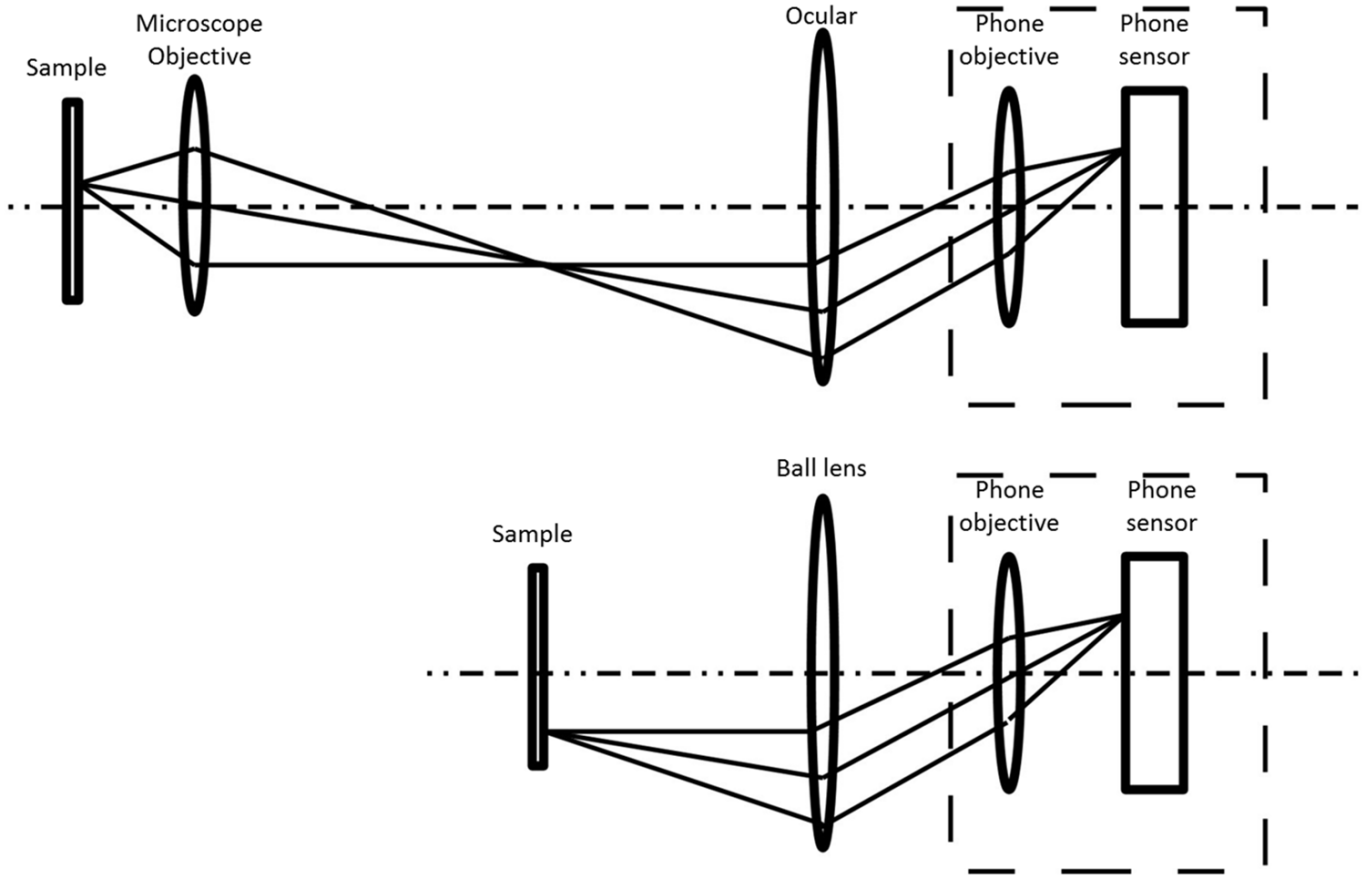
Cellphone camera coupled to classical microscope (top), and used as a tube lens coupled to external micro-objective (bottom).

The design of the mobile phone is optimized for photographic imaging, and imposes limitations to the optical scheme, when used in the microscope configuration. Fig 1 illustrates the two possible realizations of a mobile phone microscope, with the lens of the phone camera focused to infinity. In the first configuration, shown in the top of Fig 1, the phone camera replaces the human eye in the exit pupil of a classical microscope. Since the diameter of the phone lens (not to scale in the figure) is smaller than the average diameter of the pupil of the human eye, the phone lens tends to reduce the numerical aperture and the achievable resolution of the instrument. This scheme results in a rather bulky setup, as it requires a complete lab microscope to be present and properly coupled to the cellphone.

In the second configuration, shown in the bottom of Fig 1, the mobile phone lens is used as the tube lens. It allows for a very compact implementation, with external lens mounted directly to the cellphone. However, to obtain an acceptable magnification *M* ≥ 1 between the sample and the image, the external objective should have a very short focal length.

Human blood cells have disk-like shape with outer diameter in the range between 7 and 9 *μ*m. For imaging of malaria parasite, nested inside the blood cell, the optical resolution *r* should be: *r* ≤ 1 *μ*m. The corresponding minimum resolved spatial frequency is estimated as *F*_*min*_ ≥ 1/2*r* = 500 lp/mm.

The camera of a standard mobile phone has focal length in the range *F* ∼ 3…5 mm, back numerical aperture of *NA* ≤ 0.25, and the pixel pitch in the image sensor of *p* ∼ 1.25…2 *μ*m. According to the sampling theorem, one period of the maximum spatial frequency should cover at least two camera pixels:

$$\begin{array}{c} p<\frac{\lambda M}{4NA}, \end{array}$$ (1)

where *NA* is the numerical aperture. According to Rayleigh criterion, the resolution is given by:

$$\begin{array}{c} r\approx \frac{0.61\lambda }{NA}. \end{array}$$ (2)

So the minimum magnification *M* between the sample and the sensor of the phone should be at least:

$$\begin{array}{c} M>2\frac{p}{r}, \end{array}$$ (3)

where *p* is the pixel pitch, and *r* is the required resolution. Assuming *p* in the range 1.25 …2 *μ*m, and *r* ≈ 1 *μ*m, we obtain the condition:

$$\begin{array}{c} M>2.5\ldots 4. \end{array}$$ (4)

Since the focal length of the phone objective ∼ 4 mm is much smaller than the standard focal length of a tube lens, which is of the order of 200 mm, the maximum achievable magnification would be ∼ 50 times smaller than with the standard tube lens. The focal length of the objective lens, according to 3 should be rather short:

$$\begin{array}{c} F_{o}<F/M\approx 1.33\;mm. \end{array}$$ (5)

Even 100× standard microscope objective has a longer focal length, therefore glass ball lens, allowing for a very short focal lengths, is the natural choice.

References [8–10, 19, 20] mention cellphone as a promising diagnostic tool for malaria detection and exploit glass ball lenses as a cheap objective for the cell-phone based microscope. However up to date we are not aware of any practical imaging of malaria parasite with a ball-lens microscope, that allows for analysis of its morphology. It is of a great interest to perform the optimization of the optical design of the ball lens cell-phone microscope to its ultimate performance. The optimization should define the parameters, such as the material of the ball lens, the ball lens diameter, the distance from the ball lens to the phone objective, and the size and position of the aperture stop, that defines the numerical aperture and sets the diffraction limit to the achievable resolution. Optimized setup should provide the highest image quality in the widest field, with white light illumination.

For preliminary ray tracing we approximated the cellphone lens with a paraxial model. To obtain a better estimate, we used the raytracing model of a cellphone micro-objective, described by the US patent 20070024958, with focal length of *F* ≈ 4 mm and the image space numerical aperture *A* = 0.2. The realistic model of the objective allows to take into account the practically important vignetting factors. We found that for the rest, the standard phone objective has a very good correction and performs almost as good as the ideal paraxial lens. This is explained by the fact, that the numerical aperture in the image space is *M* times smaller than in the object space. Assuming maximum numerical aperture in the object space of *A* = 0.2 and magnification *M* = 4, we obtain *A*/*M* = 0.05, which corresponds to *F*# = 10, where *F*# corresponds to the photographic focal number of the cellphone lens. Modern cellphone lenses are corrected for *F*# ≤ 3, therefore they are expected to have diffraction limited quality at *F*# ≥ 10.

**Fig 2:**
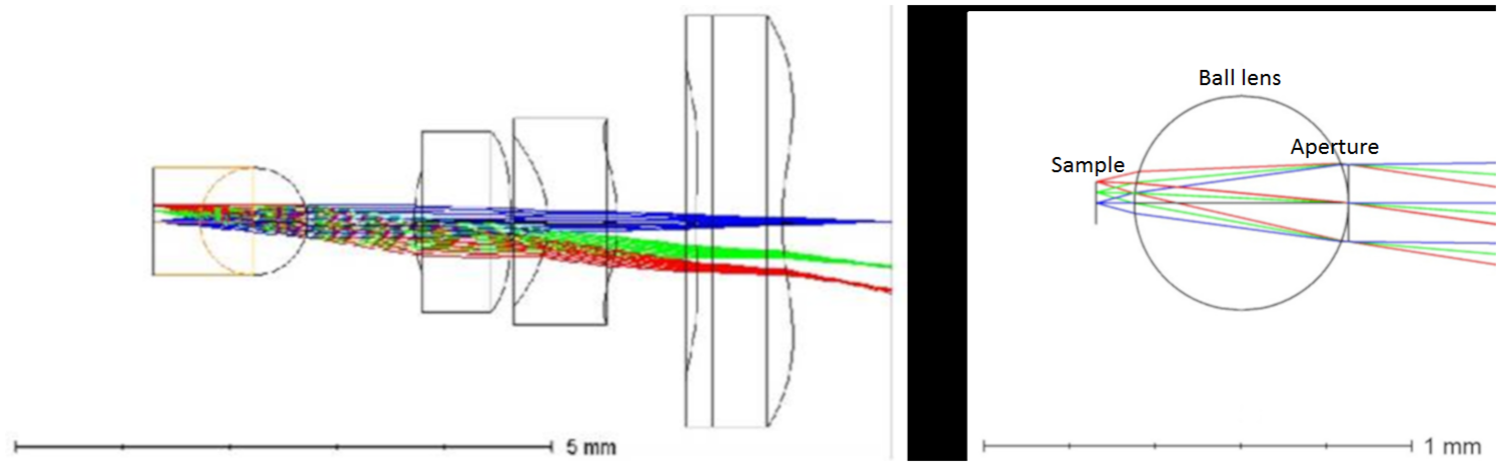
Ball lens coupled to the cellphone lens, to form a microscope and the zemax model of the aperture inserted behind the ball lens.

The optical scheme of the cellphone microscope integrated with an optimized ball lens and the designed aperture, is shown in Fig 2. Our multi-parametric optimization of the optical scheme, performed with Zemax Optics Studio, resulted in the following practical conclusions:

- The optimal object space numerical aperture of the glass ball microscope limited by the spherical aberration should not exceed ∼ 0.2, limiting the maximum achievable resolution. The optimal theoretical position of the system pupil is in the center of the ball lens. Since such a position is difficult (but not impossible) to implement, the next practically acceptable position for the aperture stop is directly behind the ball lens. The position and the size of the stop are rather critical for the image quality.
- The system is almost insensitive to the distance between the glass ball and the phone lens. In our simulation we have changed this distance in the range from 0.5 to 3 mm, without any significant change of the on-axis image quality. Longer distances result in significant vignetting in the off-axis areas, which are anyway strongly aberrated. Vignetting can be used to align the ball lens with the optical axis of the microscope objective, by centering the image circle on the cellphone screen.
- The field of view is limited by the off-axis aberrations of the ball lens, with major contribution from the field curvature.
- Due to high refraction difference between the cell tissue and air, the image of a blood cell in air has a very high contrast. This contrast is masking parasite inside the cell. Filling the space between the ball lens and the sample with immersion liquid mitigates the refractive index difference, allowing for clear imaging of the low-contrast parasite inside the cell. Immersion also reduces the amount of spherical aberration by eliminating the contribution of the front surface of the ball lens. The integral effect of immersion allows for a wider field of view. The type of immersion liquid is not of critical, as long as its refraction index is close in the range between that of water *n* ≈ 1.33 and glass *n* ≈ 1.51.
- The chromatic aberrations did not contribute significantly to the resolution loss.

To mount the ball lens on the mobile phone, we used 50 *μ*m aluminom foil. The ball lens was mounted between two pieces of foil with a double sided tape. The mounted ball lens was aligned and attached to the cellphone with a 0.0625 mm scotch tape. The front piece, facing the sample, has aperture of about 200 to 300 *μ*m, to provide for a sufficient field of view. The aperture stop was formed in the piece of foil facing the phone lens. The diameter of the aperture was chosen according to the results of optimization (see Table 1). The misalignment of the aperture stop within 10% of the aperture diameter does not constitute any serious degradation in the performance of the optical system. This is quite easily achievable in the practical implementation of the system. The glass ball was self-centered between two apertures and mounted directly on the phone. The lateral position of the ball lens assembly with respect to the phone lens was aligned by centering the defocused image circle produced by a remote extended light source.

**Table 1:** Optical parameters of the ball lens microscope, optimized for the field of 100 × 100 *μ*m, with different diameters of BK7 glass ball lens.

Ball size	Stop radius	*M*	NA	*r*_0_ *μ*m	*r*_35_ *μ*m	*r*_70_ *μ*m	*r*_*dl*_ *μ*m	*F*_*m*_ lp/mm
**0.5 air**	0.09 mm	-8.5	0.24	0.95	1.28	3.6	0.95	872
**0.5 imm**.	0.11 mm	-11.5	0.23	1	1.5	3.1	0.85	836
**1 air**	0.16 mm	-6.1	0.21	1.3	1.5	2.7	1.3	763
**1 imm**.	0.2 mm	-4.6	0.19	1.6	2.1	2.3	1.6	690

The illumination was provided by an array of white LED, supplemented by a scatterer formed by a piece of white paper. We found that the position of the scatterer is not critical as long as the angular size of the scatterer exceeds 2 *NA*, where *NA* ≤ 0.2 is the numerical aperture of the glass-ball microscope [21–23].

Table 1 illustrates the visible polychromatic performance of the optimized glass ball microscope with 0.5 mm and 1 mm lenses in air and immersion (imm). The resolution is estimated on axis *r*_0_, and in the corner of the field of 50 × 50 *μ*m and 100 × 100 *μ*m, *r*_35_ and *r*_70_, where *r* is the *rms* radius of the geometrical point spread function. Obviously, the real resolution is limited by the diffraction limit *r*_*dl*_ for any *r* < *r*_*dl*_. The theoretical cut-off spatial frequency at zero MTF contrast corresponding to the estimated numerical aperture (NA) at the wavelength λ = 550 nm is listed as *F*_*m*_ on Table 1.

We have experimentally validated the performance of the cell-phone microscope with the optical parameters prescribed in Table 1. The “Moto X-style” medium-range smart-phone used in our experiments has the following sensor specification: Sensor dimension is 5.99 × 4.5 mm with pixel size of 1.12 *μ*m. Focal length of the phone objectives is 4.61 mm and the image resolution is 5344 × 4008 pixels. We used 0.5 mm diameter, N-BK7 Ball Lens (Edmund Optics Stock #45 − 553) and 1 mm diameter N-BK7 (Edmund Optics #43 − 708). The aperture diaphragm prescribed in the model was created in the aluminum sheet using laser machine for maximum precision, and mounted directly on the back of the ball lens. To avoid uncertainty caused by the autofocus function, the camera phone was fixed to infinity. Self-timer was used to avoid any vibration due to touchscreen operation.

**Fig 3:**
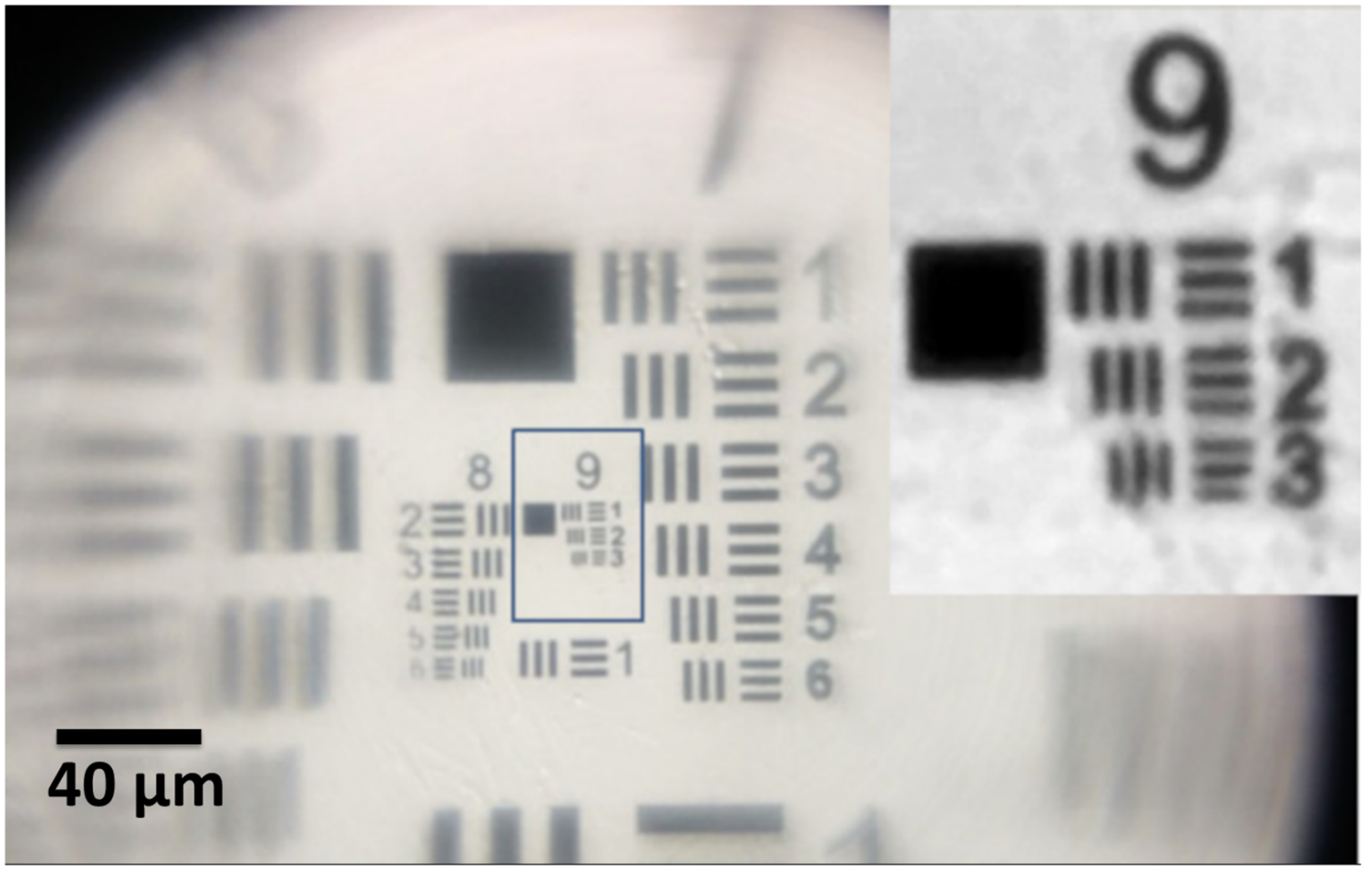
The third element of the last group of the 1951 USAF target, imaged without immersion, resolved with the optimized cellphone microscope, equipped with a properly stopped 0.5 mm ball lens. The width of the smallest resolved bar in the inset is 0.77 *μ*m, corresponding to *F*_*max*_ = 645 lp/mm.

Fig 3 shows the image of the last group of the USAF1951 resolution target (PS7P from Pyser-SGI), obtained with a 0.5 mm ball lens. The optical system clearly resolves the maximum element in Group 9 with 645.1 lp/mm, with the width of resolved bar (*r*) = 0.77 *μ*m. A 1 mm ball lens, equipped with proper aperture, also clearly resolved this group, though with smaller magnification. The malaria parasites in the ring trophozoites stage have size of about (1/5)^*th*^ of the diameter of red blood cell. Formally, the obtained spatial resolution is sufficient to detect the presence of parasite in a Giemsa stained thin blood smear. However, the parasite inside blood cell have a rather low optical contrast, therefore the practical detection of a parasite is the only ultimate criterion of the method applicability.

## Practical detection of the malaria parasite

*P. falciparum* parasites from the NF54 strain were obtained from the Radboud University Medical Center (Nijmegen, The Netherlands). Parasites were in vitro cultured as described by Marin Mogollon et al [24]. In brief, parasites were cultured using the following conditions; RPMI-1640 culture medium supplemented with L-Glutamine and 25 mM HEPES (Gibco Life Technologies) to which was added 50 mg/L hypoxanthine (Sigma). Culture medium was supplemented with 10% human serum and 0.225% NaHCO3. Parasites were cultured at a 5% hematocrit under 4% O2, 3% CO2 and 93% N2 gas-conditions at 75 rpm at 37°C in a semi-automated culture system in 10ml flasks (Infers HT Multitron and Watson Marlow 520U). Fresh human serum and human red blood cells (RBC) were obtained from the Dutch National Blood Bank (Sanquin Amsterdam, the Netherlands; permission granted from donors for the use of blood products for malaria research and microbiology test for safety). RBC of different donors were pooled every two weeks, washed twice in serum free RPMI-1640 and resuspended in complete culture medium to 50% haematocrit. Human serum of different donors were pooled every 4 to 6 months and stored at -20°C until required. From the in vitro culture, thin blood smears were prepared of mixed infected Red Blood Cells (RBCs), early ring- to late schizont-stage parasites, slides were fixed in 100% methanol and stained with a 4% Giemsa staining to visualize the parasites of the blood-stage cycle as described in Janse et al [25].

**Fig 4:**
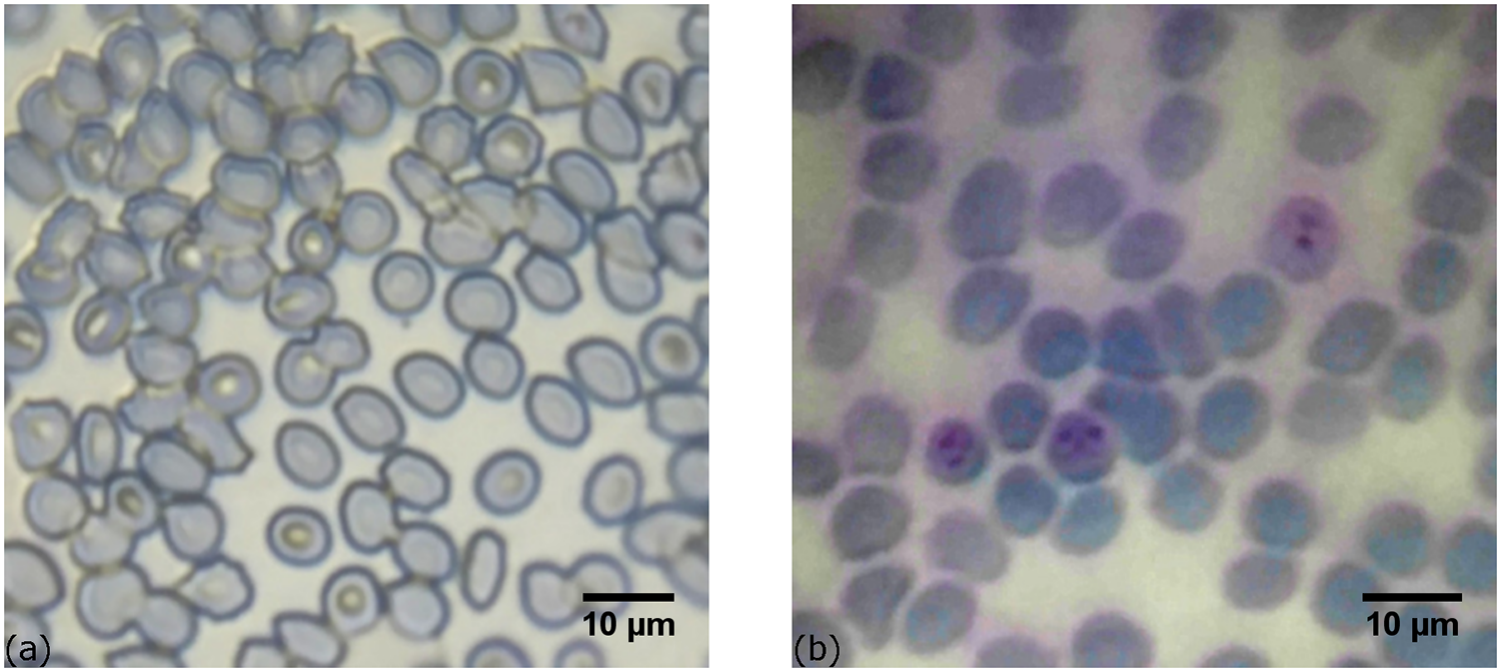
Images of *in vitro* cultured *P. falciparum* parasites in Giemsa-stained thin blood smears taken with 0.5 mm ball lens cell-phone microscope registered without immersion oil, with high contrast masking the cell contents (a) and with immersion revealing parasites inside blood cells (b).

Giemsa stained thin blood smears were examined using a cell-phone equipped with a 0.5 mm, NBK-7 ball lens with and without oil immersion as described in Table 1. In the dry imaging, the light is mostly scattered on cell-to-air boundaries, resulting in cell imaging with high contrast, while the malaria parasites contained within the blood cells are hardly detectable as shown in the left of Fig 4.

**Fig 5:**
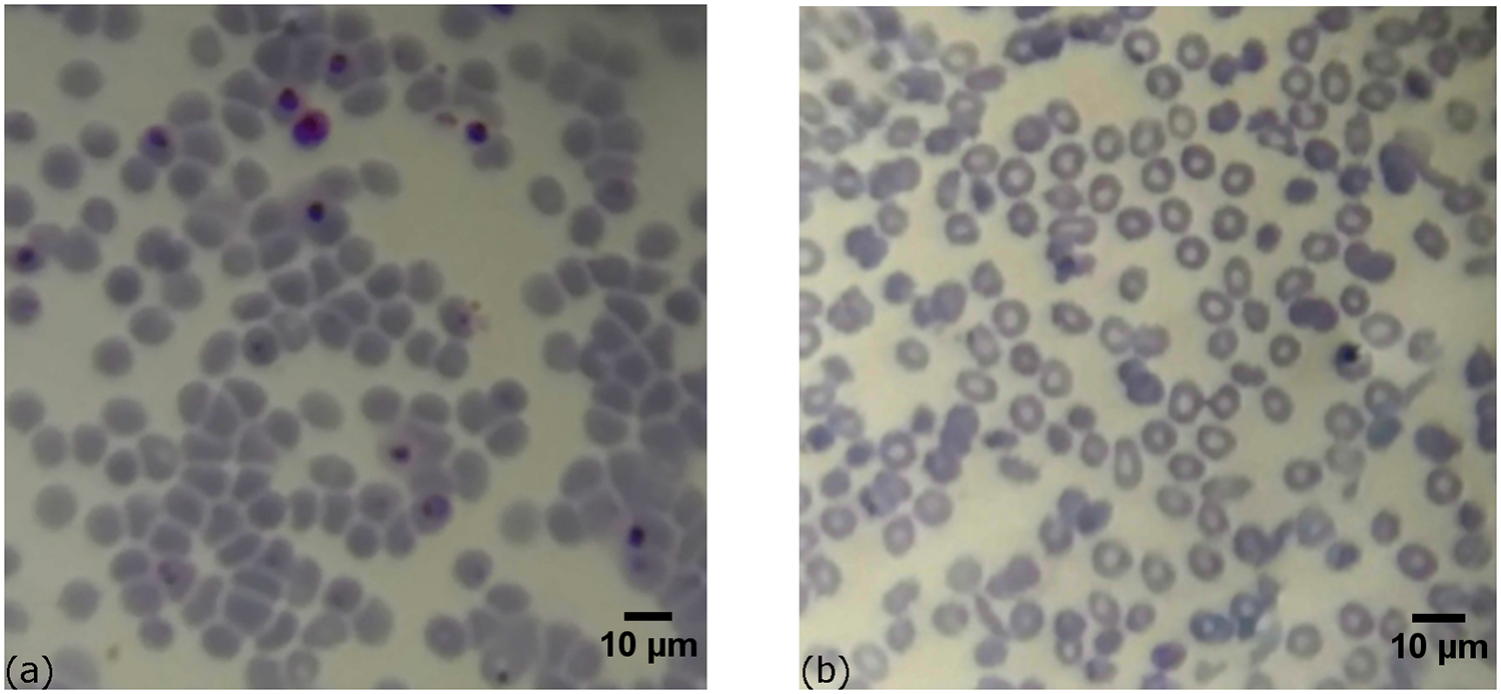
Images of Giemsa-stained thin blood smears with *in vitro* cultured *P. falciparum* parasites taken with 1 mm ball lens cell-phone microscope (a), and non infected red blood cells (b).

Immersion in oil with refractive index (n = 1.518) reduces the refraction and visibility of the cell-to air interfaces and improves the relative visibility of the cell contents. Fig 5 is an image of *in vitro* cultured *P. falciparum* parasites in Giemsa-stained thin blood smear taken with 1 mm ball lens cell-phone microscope using immersion oil. The *in vitro* blood sample contains infected red blood cells with parasites at different points of development. After staining, infected red blood cells are clearly visible including those containing very mature parasites (schizonts). System magnification is 4× and an increased field of view of ∼ 150 *μm* is realized. This is an obvious gain as compared to the 100 *μ*m field obtained with the optimized 0.5 mm ball microscope. Although this optimized design model is sufficient for the detection of *P. falciparum* infected red blood cells, the 4× magnification is, however, not sufficient for distinct discrimination of the morphology of the parasite.

To validate the presence of the parasites in the acquired images we conducted microscopic examination of healthy blood sample, subjected to the same fixation and staining process as the infected sample. An image obtained using this system configuration is shown in the right side of Fig 5. The difference between the infected and the healthy samples is clearly visible.

The number of total fields required for a relevant diagnosis is determined by the field of view of the microscope. In the gold-standard case, it is approximately 180 × 180 *μ*m for 1.25 *NA*, 100 × oil immersion microscope objective. Current WHO standard requires 100 fields of view to provide the final determination of malaria infection. The standard limit of detection is 30 parasites/*μ*l of blood.

The practically realized field of view of 100 × 100 *μ*m implies that a larger number of fields will be taken for proper diagnosis, as compared to the current standard. Considering the gains of mobility and simplicity, ball lens microscope still remains a usable tool for field diagnostics, where little or no diagnostic tool is available. The mobile phone can be used not only for imaging, but also for control and automation of the sample stage movement, providing automatic registration and pre-processing of a large number of images.

**Fig 6:**
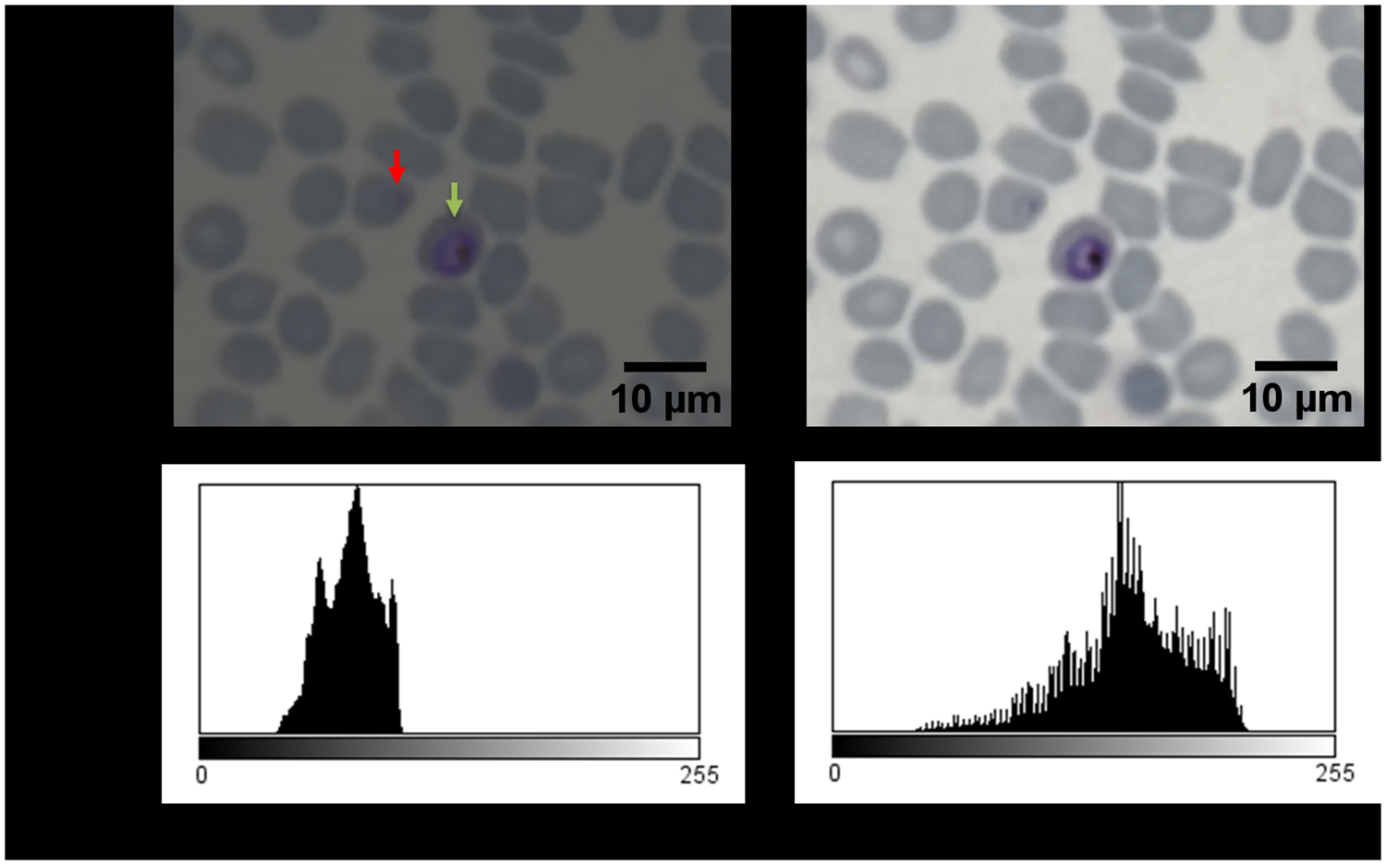
Images of *in vitro* cultured *P. falciparum* parasites in Giemsa-stained thin blood smears taken with 1mm ball lens cell-phone microscope using 4x digitally zoom. Visualizing an early ring stage trophozoite (red arrow) and a matured trophozoite (green arrow). Raw data from cell-phone microscope (a). HDR mode (b).

The contrast of the parasite images obtained with oil immersion is rather low as compared to results obtained from standard light microscopy, therefore post-processing algorithms that enhance the visual image contrast by extending the histogram to available dynamic range, are of great practical value. Fig 6 shows the gain in contrast, obtained by registering the cellphone images in HDR mode. Since the HDR mode combines information from a number of images, the HDR mode improves both the visibility and the information contents of the image. In this particular case, it facilitates the detection of the early ring trophozoites.

**Fig 7:**
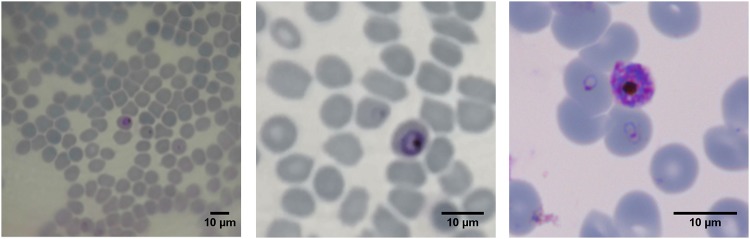
Images of *in vitro* cultured *P. falciparum* parasites in Giemsa-stained thin blood smears. Taken with 0.5 mm ball lens cell-phone microscope (left). Taken with 0.5mm ball lens cell-phone microscope, with applied 4× digital zoom and HDR mode (middle). Image of *in vivo* human *P. falciparum* infection taken by light microscope, ×60 objective obtained using a high-end Zeiss Light microscope in Leiden University Malaria group laboratory (right).

Fig 7 compares the HDR and normal images. The right image is the reference, acquired using standard high resolution bright field microscope with 1.25 *NA*, ×60 magnification and oil immersion, obtained using a high-end Zeiss Light microscope in Leiden University Malaria group laboratory. The High Dynamic Range (HDR) imaging is especially useful for improved detectability with human operator. No other image manipulation was performed beyond reported.

**Fig 8:**
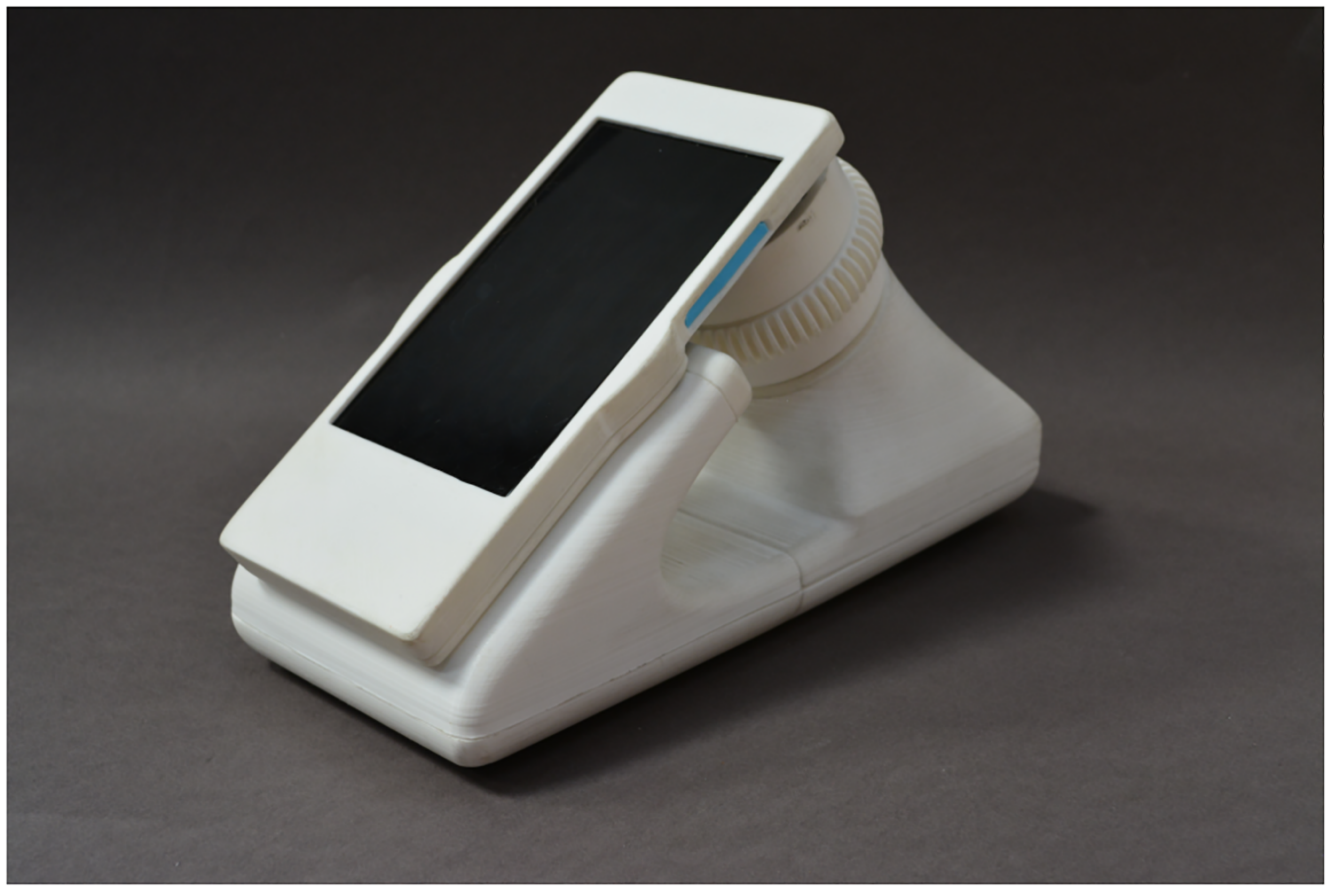
Motorized prototype with automated x-y movement of blood sample, which enables fast acquisition of large number of images.

**Fig 9:**
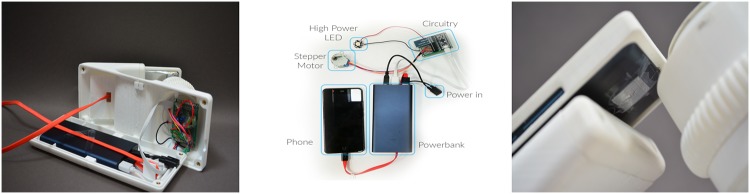
Overview of the system design (a) depicts the battery back up and micro-controller used for the digital control of the stepper motor (b) shows the circuitry while (c) depicts the attachment of the ball lens mounted in a piece of aluminum foil and attached to the smart-phone with a scotch tape.

The limited field of view requires a large number of images to be acquired for a reliable test outcome. To automate the procedure, we have built a simple device with an automated x-y translation stage (see Figs 8 & 9), that allows for motorized movement and easy manual image focusing of the blood smear sample. The stage is printed on a 3D printer and provides sufficient precision for acquiring a large number of sharp images over a significant field. Our device is integrated with a single motor which provides a circular x-y movement of the blood sample. Although this rotational movement of the sample is uncommon in medical practices, we however confirmed with stakeholders that this is a viable solution since no field of view is repeated twice. This sample movement technique considerably reduces the size, price and power consumption of our device. A stepper motor 28BYJ-48 5V (with a unit cost of 1 euro) is integrated into the device using a low-cost micro-controller which cost approximately 10 €. An SP 10000 mAh Multi USB battery which cost 13 € provides a power back-up for situations of sudden power outages as common in remote settings. Total low volume cost which also includes the cost of 3d printed parts, illumination, ball lens holder, diffusers, screws and magnets used for the prototype is estimated at approximately 40 €. This price estimation excludes the cost of the integrated smart-phone. We expect that bulk production will imply a significant reduction in cost price. With image acquisition rate estimated to be in the range of 1-6 seconds per frame, diagnosis of patient sample could be completed in less than 30 minutes. The live demonstration of the system is available at: https://www.youtube.com/watch?v=jnzKhMNlSiE&feature=youtu.be.

## Conclusion

Cellphone based microscope with a ball lens objective has been optimized for high resolution bright field imaging of malaria parasite in thin blood smears. Parasites in various stages of infection have been detected in sample infected smears. We found that the system based on the 0.5 mm glass ball lens using immersion enables morphological identification of the parasite, which is critical to accurate interpretation of the test results. It offers high spatial resolution, high system magnification (8.5×) in a reduced field. The optimized system based on a 1 mm ball lens however offers a larger field of view of about 150 *μm* and lower magnification (∼ 4.5×), which is useful for preliminary detection. The performance has been critically analyzed with respect to optimal numerical aperture, field of view, camera pixel pitch, the system magnification, lens size, and immersion. Compared to the previously reported systems [8–10, 19, 20], we have significantly improved the resolution of ball-lens cellphone-based microscope system. Use of immersion is instrumental for morphology identification as it allows for resolving the low-contrast contents of infected cells and reduces the field curvature, thus extending the field of view. Giemsa staining protocol is well simplified and can be implemented on the field by rural health field workers, patent medicine vendors etc. and the process of fixing with methanol is also realizable on the field. Our user design interaction interface survey with potential users and stakeholders on the field in Nigeria shows the potential of integrating our device with lab on the field diagnostic method.

## Supporting information

S1 Video 3-D stage video demonstration. The live demonstration of the system is available at https://www.youtube.com/watch?v=jnzKhMNlSiE&feature=youtu.be.
(MP4)
